# miR-29a regulated ER-positive breast cancer cell growth and invasion and is involved in the insulin signaling pathway

**DOI:** 10.18632/oncotarget.15928

**Published:** 2017-03-06

**Authors:** Zhi-hua Li, Qiu-yun Xiong, Liang Xu, Peng Duan, Qianwen Ou Yang, Ping Zhou, Jian-hong Tu

**Affiliations:** ^1^ Prevention and Cure Center of Breast Disease, The Third Hospital of Nanchang City, Key Laboratory of Breast Diseases in Jiangxi Province, Nanchang, JiangXi 330009, People's Republic of China; ^2^ Department of Endocrinology, The Third Hospital of Nanchang City, Nanchang Key Laboratory of Diabetes, Nanchang, JiangXi 330009, People's Republic of China; ^3^ Pathology Department, The Third Hospital of Nanchang City, JiangXi Breast Specialist Hospital, Nanchang, JiangXi 330009, People's Republic of China

**Keywords:** MiR-29a, breast cancer, insulin signaling pathway, cell proliferation, invasion

## Abstract

Increasing amounts of evidence show that insulin can activate different insulin signaling pathways to promote breast cancer growth and invasion. miR-29a plays crucial roles in decreasing glucose-stimulated insulin secretion, as well as in regulating breast cancer cell proliferation and EMT. However, the mechanism responsible for the regulatory effects of miR-29a on breast cancer growth and invasion and the relationship between these effects and insulin signaling remains unclear. Herein, we showed that human insulin increased miR-29a expression in ER-positive breast cancer cells and that miR-29a facilitated the ability of insulin to promote breast cancer cell proliferation and migration. We found that miR-29a-induced cell proliferation and metastasis acceleration occurred primarily through ERK phosphorylation. The IGF-1R is the upstream target gene of miR-29a, while CDC42 and p85α are the downstream target genes of miR-29a. These results have provided us with information regarding the molecular mechanisms by which hyperinsulinemia promotes breast cancer occurrence and development and thus leads to a poor prognosis in breast cancer patients and indicate that miR-29a plays an important role in breast cancer development and invasion.

## INTRODUCTION

Insulin is a multifunctional protein hormone that not only regulates the metabolic processes of many cells but can also regulate cell growth and differentiation [1]. Some studies have found that insulin plays an important role in carcinogenesis and breast cancer development by promoting mitosis or resistance to apoptosis or by affecting the sex hormone environment *in vivo* [2, 3]. Goodwin PJ et al. found that the fasting insulin level is associated with distant tumor recurrence and death in women with early breast cancer and that high fasting insulin levels are an indicator of a poor prognosis in women with breast cancer [4]. Metformin inhibits mammalian target of rapamycin-dependent translation initiation in breast cancer cells [5]. The results of a case-cohort study suggested that hyperinsulinemia is an independent risk factor for breast cancer [2]. Insulin and insulin receptor (IR) α subunit binding activate insulin receptor substrates. Because of its ability to bind different substrates, insulin can activate different insulin signaling pathways (such as the phosphatidylinositol 3-kinase/AKT kinase (PI3K/Akt) pathway or RAF kinase/mitogen activated protein kinase (Ras-MAPK pathway)) to promote breast cancer growth and invasion [3]. Regarding estrogen receptor (ER)-positive breast cancer, the tumorigenic properties of estrogen are regulated by ERα. Insulin-like growth factors (IGFs) can activate the ER, and crosstalk between insulin-like growth factor 1 receptor (IGF-1R) and ER signaling exists in breast cancer [6]. Wairagu PM et al. found that insulin exerts priming effects on estradiol-induced breast cancer metabolism and growth. These findings suggest that ER activation under chronic hyperinsulinemic conditions increases breast cancer growth through cell cycle and apoptotic factor modulation and nutrient metabolism and provide mechanistic evidence indicating that metformin has beneficial effects in ER-positive breast cancer patients with diabetes and may be used as a treatment in such patients [7]. Obesity promotes greater ERα-positive breast cancer cell viability and growth by enhancing the crosstalk between nongenomic ERα signaling and the PI3K/Akt and MAPK pathways [8].

miR-29a is the predominant member of the miR-29 family, which has multiple target genes and plays crucial roles in various biological processes, including cellular proliferation, differentiation, development and apoptosis [9]. miR-29a expression was up-regulated in the serum of patients with type 2 diabetes and in 3T3-L1 adipocytes cultured with high insulin and high glucose [10]. miR-29a expression was also up-regulated in the serum and tissue of breast cancer patients [11, 12] but was down-regulated in breast cancer cells [13]. Several studies have confirmed that miR-29a regulates breast cancer cell EMT and metastasis by inhibiting tristetraprolin expression [14]. Given the findings of the above studies, we hypothesized that miR-29a may be an important endogenous molecule in insulin-mediated promotion of breast cancer cell growth and invasion.

This study aimed to explore the mechanism by which miR-29a regulates breast cancer growth and invasion via the insulin signaling pathway to elucidate the molecular mechanism by which hyperinsulinemia promotes breast cancer occurrence and development, thereby leading to a poor prognosis in breast cancer patients, and to determine the important role of miR-29a in breast cancer development and invasion.

## RESULTS

### Establishment of breast cancer cell models with high insulin

**Figure 1:**
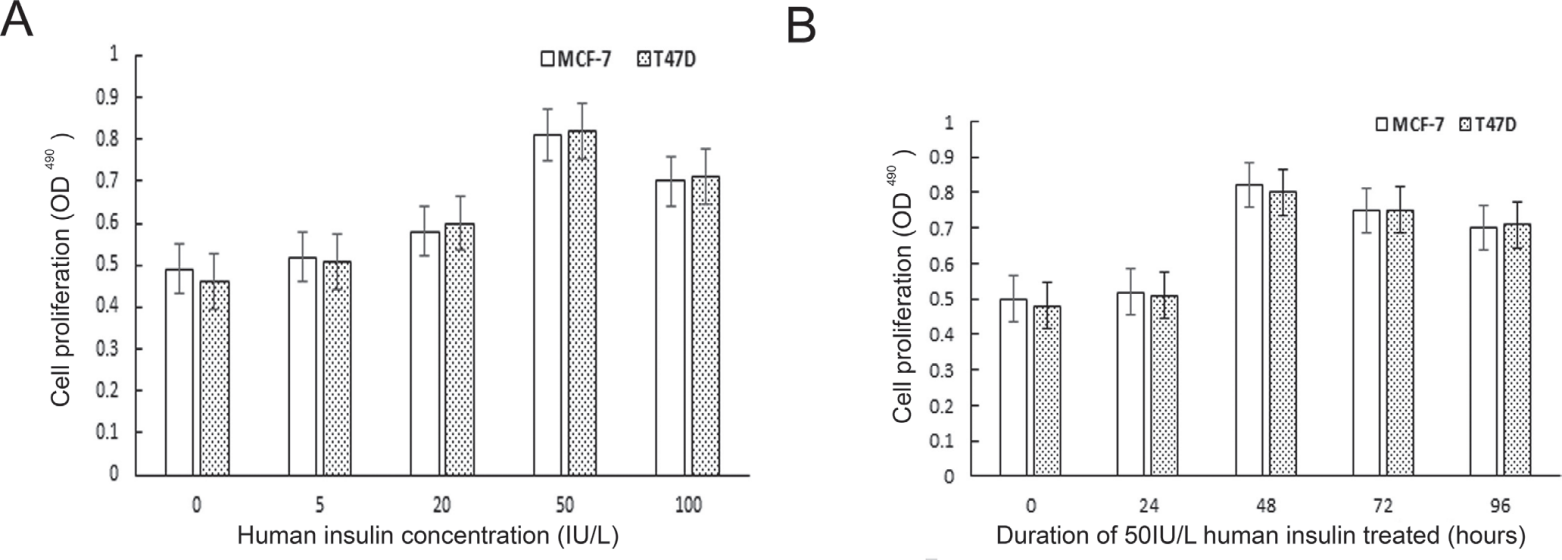
The effect of human insulin on ER-positive breast cancer cell proliferation (**A**) The best concentration for the maximum rate of cell proliferation is 50 IU/L. (**B**) The best time of action for the maximum rate of cell proliferation is 48 h.

The proliferation kinetics of MCF-7 and T47D cells were detected by MTT assay, the results of which showed that all the concentrations of human insulin used herein (5.0 IU/L, 20 IU/L, 50 IU/L, and 100 IU/L) promoted MCF-7 and T47D breast cancer cell proliferation compared with the control treatment. In addition, the results of the above assay showed that stimulatory effects of insulin were dose dependent. Moreover, the results showed that different durations of human insulin treatment (0 h, 24 h, 48 h, 72 h, and 96 h) exerted stimulatory effects of different magnitudes. The breast cancer cell models in high-insulin cultures were established by identifying the insulin concentration in which and time at which the maximum rate of cell proliferation occurred. Our study showed that the maximum rate of cell proliferation occurred in cells incubated with 50 IU/L insulin for 48 h (Figure 1).

### High insulin up-regulated miR-29a expression in ER-positive breast cancer cells

**Figure 2:**
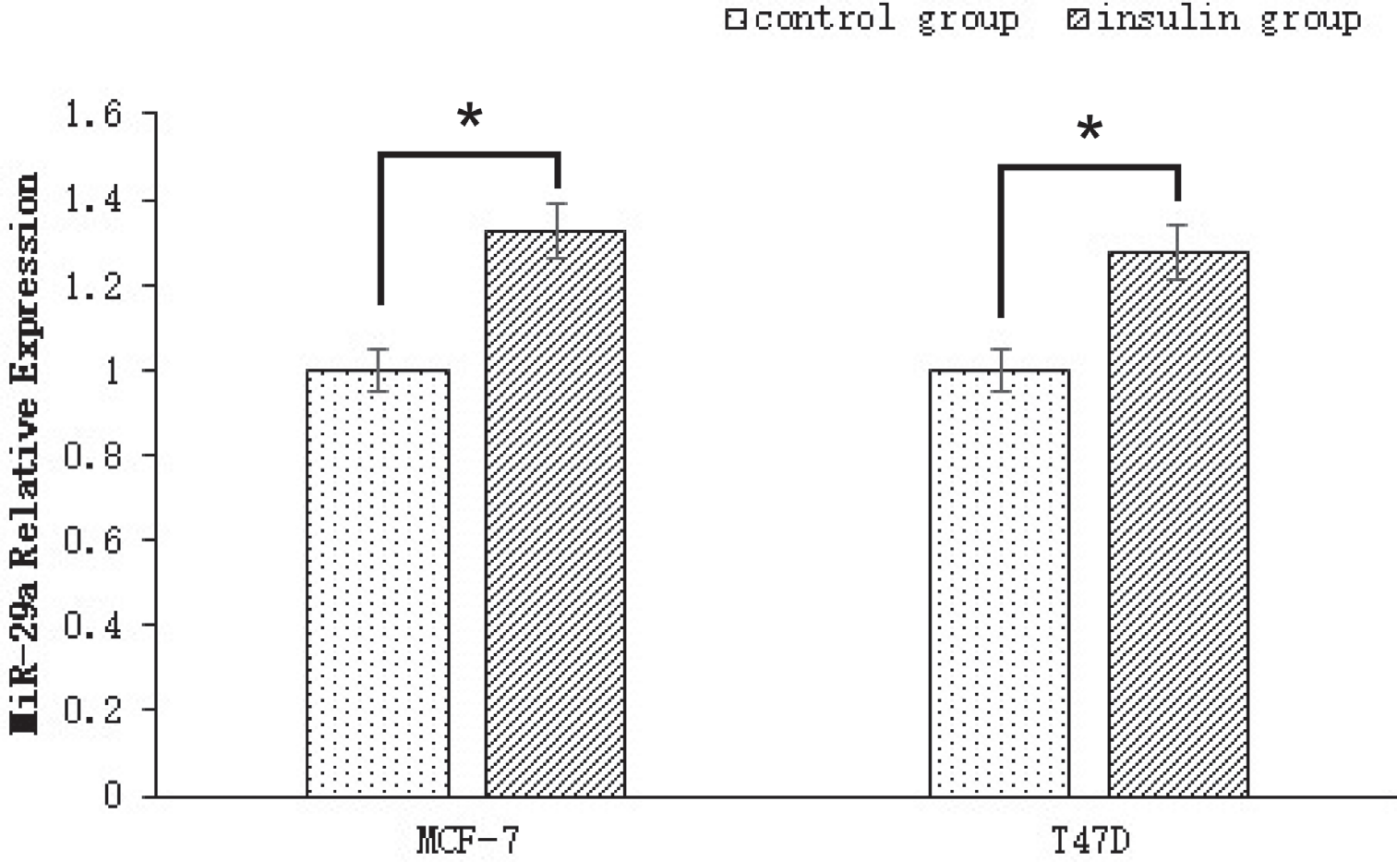
miR-29a expression in ER-positive breast cancer cells treated with human insulin miR-29a expression in the insulin treatment groups (50 IU/L of insulin for 48 h) was up-regulated by 1.325 ± 0.132 and 1.275 ± 0.113 fold compared with that in the control groups.

To explore the effect of human insulin on miR-29a expression in the ER-positive breast cancer cell lines MCF-7 and T47D, we detected miR-29a expression in both the control group and the insulin treatment group (50 IU/L of insulin for 48 h) by qRT-PCR. miR-29a expression was significantly higher in the insulin treatment groups than in the control groups (*p* < 0.001, *n* = 3), as miR-29a expression was up-regulated by 1.325 ± 0.132 and 1.275 ± 0.113 fold in the treatment groups compared with the control groups (Figure 2).

### Effect of miR-29a on ER-positive breast cancer cell proliferation

**Figure 3:**
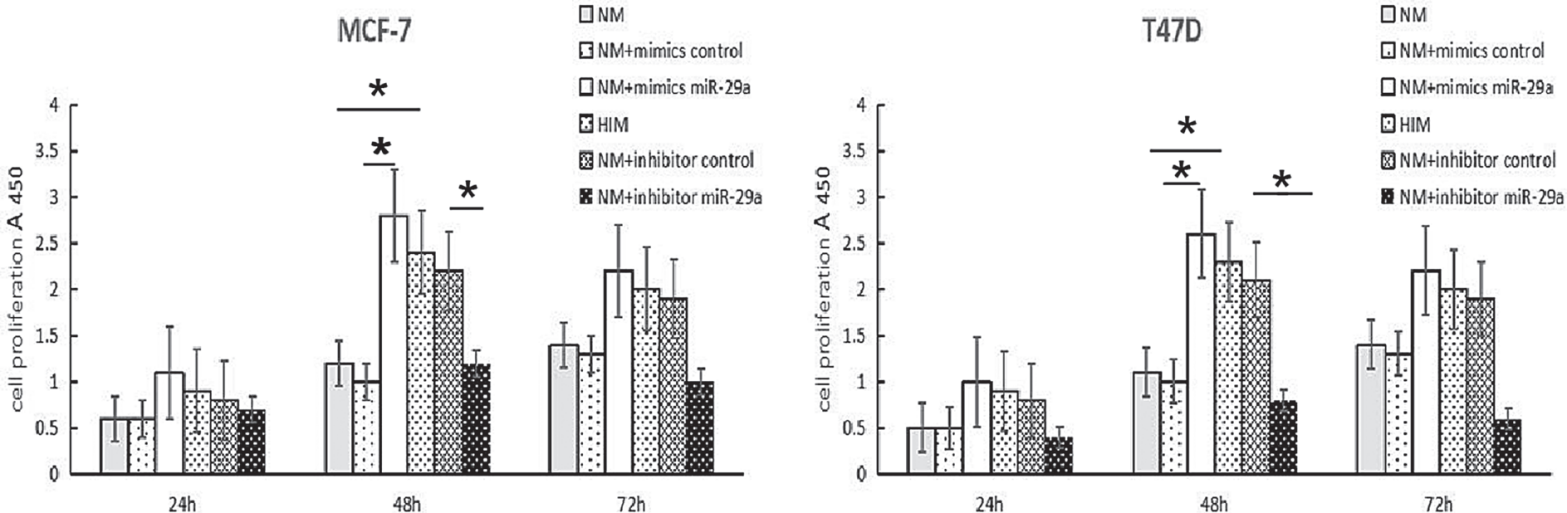
Effects of miR-29a on ER-positive breast cancer cell growth and cell cycle progression NM: MCF-7 cells with normal medium. HIM: MCF-7 cells with high-insulin medium. The results of the CCK8 assays, which were performed following transfection of the miR-29a mimic and miR-29a inhibitor into MCF-7 cells for 24-h, 48-h and 72-h time periods. The values are the mean and SD in optical density (OD) units. A representative result of 3 independent experiments is shown. *p < 0.05.

miR-29a expression levels were correlated with the degree of differentiation, which was partly dependent on cell proliferation. Therefore, we assessed the effects of miR-29a on MCF-7 and T47D cell proliferation by performing gain- and loss-of-function experiments. In these experiments, MCF-7 and T47D cells were transfected with a mimic or inhibitor and the corresponding scrambled sequence control. The cell proliferation suppressive effects of miR-29a were evaluated by CCK-8 assay, which was performed on cells in the miR-29a-mimic group, miR-29a-inhibitor group, mimic control group and inhibitor control group. As shown in Figure 3, transfection with the miR-29a mimic promoted MCF-7 and T47D cell growth and proliferation, and transfection with the miR-29a inhibitor inhibited cell growth and proliferation.

### Effect of miR-29a on ER-positive breast cancer cell invasion

Insulin promotes breast cancer cell proliferation and migration through the extracellular-regulated kinase (ERK) pathway [15]. miR-29a increases breast cancer EMT and metastasis through different molecular mechanisms [12, 16, 17].

**Figure 4:**
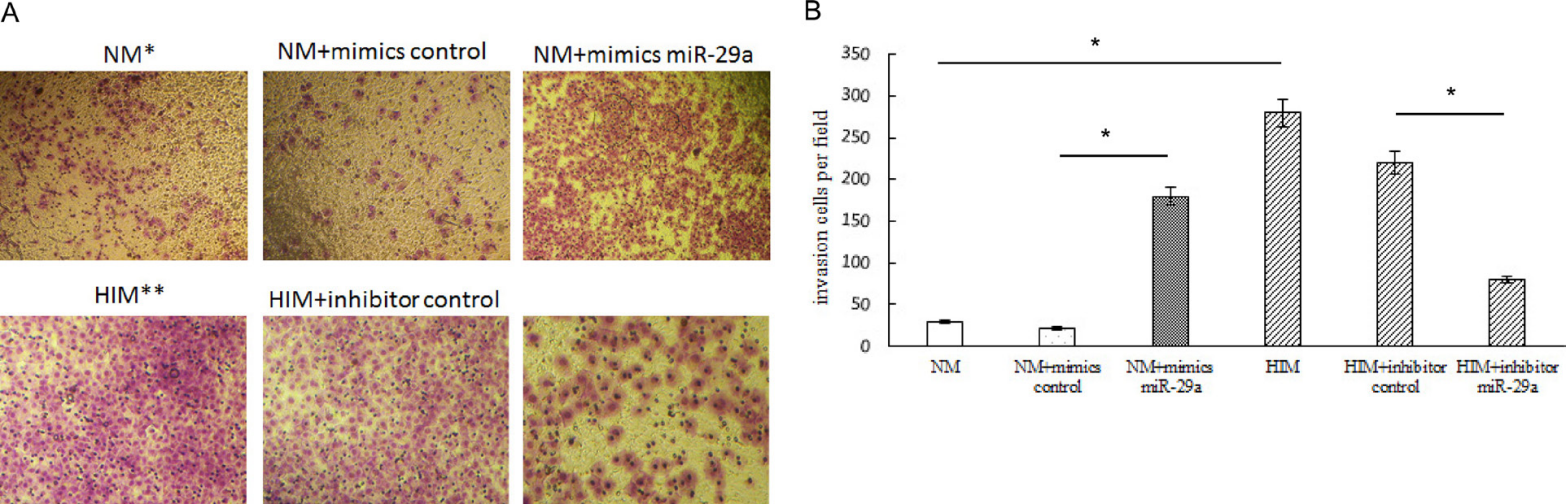
Effect of miR-29a on ER-positive breast cancer cell invasion NM: MCF-7 cells with normal medium. HIM: MCF-7 cells with high-insulin medium. (**A**, **B**) Transwell migration and invasion assays showing the effects of insulin and miR-29a over-expression or knockdown on MCF-7 cell migration and invasion activity. The migratory and invasive breast cancer cells that grew on the lower surface were stained and counted manually using a microscope (original magnification 50×) at 24 h after reseeding. Representative images are shown in the left panel. The mean number of cells per visual field was determined in four randomly selected visual fields per chamber, and the experiments were performed in triplicate (right panel). *p < 0.05.

To elucidate the mechanism underlying the relationship among insulin, miR-29a and the promotion of ER-positive breast cancer cell invasion, we evaluated transfected cell migration and invasion potential by Transwell assay (Life Technologies), the results of which showed that human insulin and the miR-29a mimic promoted MCF-7 cell invasion, while the miR-29a inhibitor reversed human insulin-induced MCF-7 cell invasion (Figure 4).

### miR-29a regulated ER-positive breast cancer cell growth and invasion and participates in the insulin signaling pathway

Insulin promotes breast cancer growth and invasion by activating the following two major signaling pathways: the PI3K/AKT pathway and the RAF/MAPK pathway [3]. p85α is the regulatory subunit of PI3K, which mediates cAMP-PKA- and insulin-induced biological effects on MCF-7 cell growth and motility [18]. AMPK, ERK1/2, and CDC42 are three key members of the MAPK signal transduction pathway, which has proliferative effects and regulates IR expression. The IR mediates and amplifies the insulin signaling pathway [19]. Tetrameric IGF-1R consists of two identical α and two identical β subunits. Ligand binding to and subsequent phosphorylation of IGF-1R triggers activation of the following two major signaling cascades via insulin receptor substrate 1 (IRS-1): the PI3K/AKT pathway and the RAF/MAPK pathway, which stimulate proliferation and facilitate protection from apoptosis [20].

**Figure 5:**
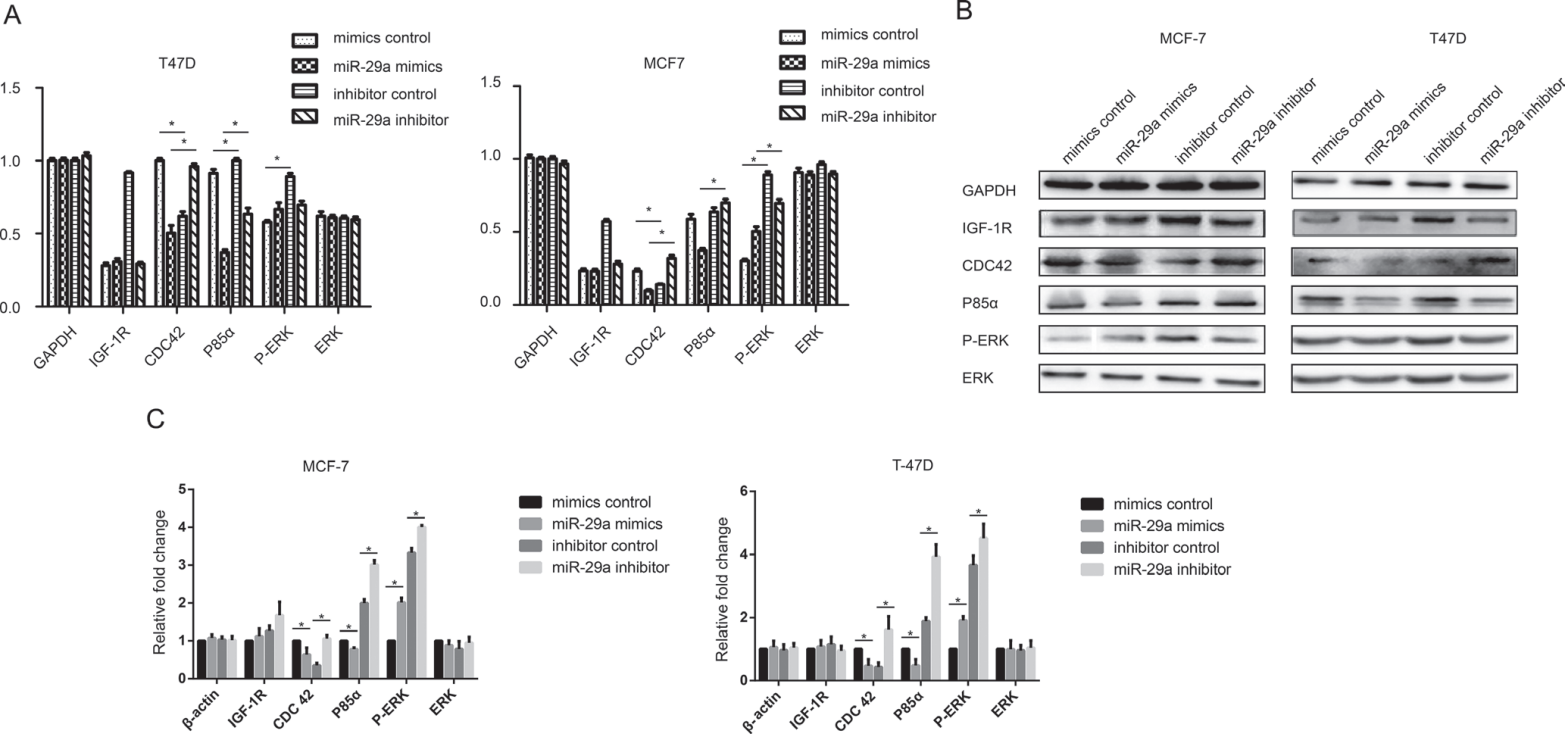
Effect of miR-29a on the insulin signaling pathways (**A**) IGF-1R, CDC42, p85α, p-ERK and ERK mRNA levels were determined by qPCR in ER-positive cells transfected with a miR-29a mimic or miR-29a inhibitor. (**B**, **C**) Western blotting analysis was performed to monitor IGF-1R, CDC42, p85α, p-ERK and ERK expression in ER-positive cells transfected with a miR-29a mimic or miR-29a inhibitor. The results showed that ERK, CDC42 and p85α are the target genes of miR-29a; however, miR-29a promotes breast cancer cell growth and proliferation mainly by activating ERK phosphorylation. *p < 0.05.

To investigate the effect of miR-29a on the insulin signaling pathway in breast cancer cells, we examined the expression of several proteins mentioned above in cells transfected with a miR-29a mimic or inhibitor. In miR-29a mimic-transfected cells, p-ERK expression was clearly up-regulated, CDC42 and p85α expression was clearly down-regulated, and IGF-1R and ERK expression was not significantly changed. However, in miR-29a inhibitor-transfected cells, p-ERK expression was down-regulated, and CDC42 and p85α expression was up-regulated, but IGF-1R and ERK expression was still not significantly altered (Figure 5A, 5B). These results show that p-ERK, CDC42, and p85 are target genes of miR-29a; however, miR-29a promotes breast cancer cell growth and proliferation mainly by activating ERK phosphorylation.

### IGF-1R knockdown can decrease miR-29a expression in breast cancer cells

**Figure 6:**
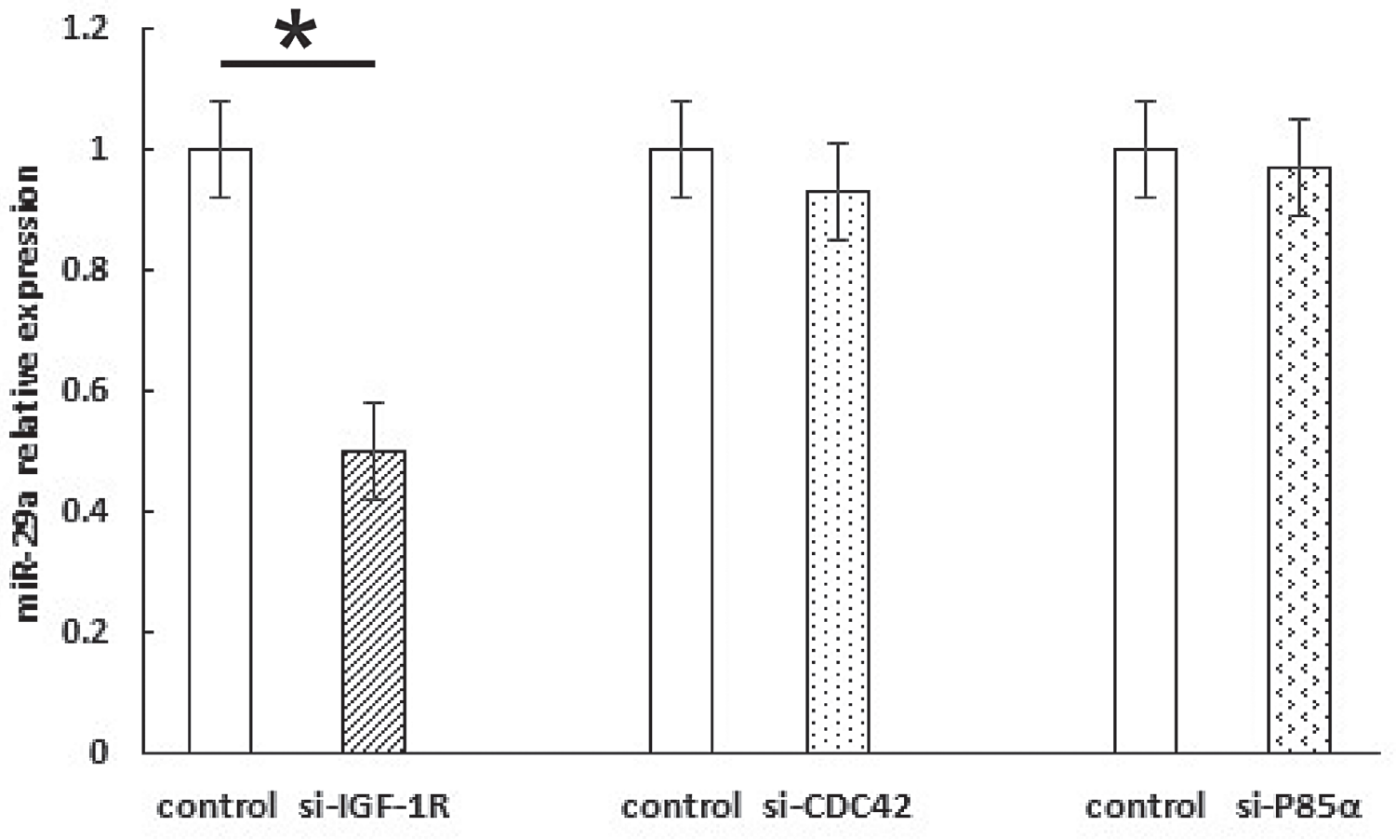
Knocking down IGF-1R can decrease miR-29a expression in breast cancer cells The results show that miR-29a expression was down-regulated by IGF-1R-siRNA but not p85α-siRNA or Cdc42-siRNA. *p < 0.05.

The IGF-1R is a tyrosine kinase cell surface receptor involved in regulating cell growth and metabolism. IGF-IR signaling pathway activation promotes breast cancer cell proliferation, survival, and metastasis [21]. Shuming Gao et al. found that the insulin-like growth factor 1 (IGF1) 3′UTR functions as a ceRNA to promote angiogenesis by sponging the miR-29 family in osteosarcoma [22]. miR-29 family members (miR-29a, miR-29b and miR-29c) negatively regulate p53 by directly suppressing p85α (the regulatory subunit of PI3 kinase) and CDC42 (a Rho family GTPase) [23]. To test whether the regulation of miR-29a in breast cancer cells cultured in human insulin medium was directly related to the IGF-1R, CDC42, or p85α, we knocked down the IGF-1R, CDC42, and p85α using siRNA. The results of this experiment showed that miR-29a expression was down-regulated by IGF-1R-siRNA and was not affected by p85α-siRNA or Cdc42-siRNA Figure 6. Based on these findings, we confirmed that IGF-1R is the upstream target gene of miR-29a, while CDC42 and p85 are the downstream target genes of miR-29a.

## DISCUSSION

A large number of epidemiological studies have found that the blood insulin levels or fasting C peptide levels of breast cancer patients were significantly higher than those of individuals in the normal population [24]. High insulin levels in the breast cancer population increase the risk of disease recurrence, and hyperinsulinemia is considered a breast cancer-independent risk factor for disease development [4, 25]. Insulin is an important molecule in metabolism, and insulin and the insulin signaling pathway play an important role in breast cancer occurrence and progression. Many previous studies have confirmed that insulin can promote breast cancer cell growth and proliferation [26, 27]. The primary mechanism underlying these phenomena involves the binding of insulin to the IR and the subsequent activation of the corresponding downstream signaling pathway. This study also demonstrated that human insulin can promote MCF-7 and T47D breast cancer cell proliferation in a dose- and time-dependent manner, findings consistent with those reported in the literature [27, 28]. These findings may be attributed to the fact that although aberrant over-expression of the IR in breast cancer cells significantly increases the effects of insulin on such cells, IR over-expression causes a certain degree of insulin receptor saturation on the cell membrane. When the amount of insulin bound to the receptor reaches a particular threshold, the stimulatory effects of human insulin on breast cancer cells no longer increase. Therefore, we determined the concentration at which and the time period during which insulin induced the maximum rate of cell proliferation, as demonstrated by MTT assay. We subsequently established a cell model that involved breast cancer cells cultured in 50 IU/L human insulin medium for 48 hours. This model was used during follow-up experiments.

MicroRNAs are a class of small, single-stranded non-coding RNAs that play crucial roles in various biological processes, including cellular proliferation, differentiation, development and apoptosis [29]. miR-29a is a conserved miRNA that acts as an oncogene, is highly expressed in breast cancer [12], and plays a major role in type 2 diabetes by participating in the insulin signaling pathway, as well as in insulin resistance [30]. In our study, miR-29a expression in insulin-treated MCF-7 and T47D cells was up-regulated by 1.325 ± 0.132 and 1.275 ± 0.113 fold compared to its expression in control MCF-7 and T47D cells. These results showed that insulin promotes miR-29a expression in breast cancer cells. However, it is not clear whether miR-29a is involved in the regulatory mechanisms by which insulin promotes breast cancer cell proliferation and invasion.

To explore the possibility that miR-29a affects cell proliferation and invasion, we performed gain- and loss-of-function experiments on our cell model. In this study, a miR-29a mimic promoted the proliferation of MCF-7 and T47D breast cancer cells, and a miR-29a inhibitor depressed the proliferation of these cells. Interestingly, previous studies have yielded controversial results regarding the effects of miR-29a on cell proliferation [12, 13, 31]. For example, Zhong S et al. found that miR-29a expression was up-regulated in drug-resistant cells and that miR-29a participated in drug-resistance by targeting PTEN to inhibit cell apoptosis [32]. Choghaei E et al. reported that miRNA-29a inhibitors promoted Taxol-induced apoptosis in MCF-7 breast cancer cells [33]. Pei YF et al. found that up-regulation of miR-29a accelerated cell proliferation, while down-regulation of miR-29a inhibited cell growth [12]. However, Wu Z et al. [13] proposed that miR-29a expression was down-regulated in breast cancer cells. miR-29a arrested cells at G0/G1 phase and suppressed tumor growth by down-regulating B-myb. This controversy may be attributed to the fact that miR-29a may have different regulatory functions depending on its environment and that it may play different roles in different diseases by initiating different signal transduction pathways.

Previous studies reported that insulin and miR-29a can promote breast cancer invasion and metastasis. Insulin increased MCF-7 human breast cancer cell proliferation and migration via the ERK pathway [15]. miR-29a negatively regulates the EMT regulator N-myc-interactor, which upregulates the mesenchymal phenotype of breast cancer cells and promotes tumor invasion [34]. miR-29a can suppress tristetraprolin [14] and can alter CXCR4 mRNA stability and CXCR4 protein expression [17], thereby increasing breast cancer EMT and metastasis. miR-29a promotes cell proliferation and EMT in breast cancer by targeting ten-eleven translocation 1 [12]. We found that human insulin and a miR-29a mimic promoted MCF-7 cell invasion, while a miR-29a inhibitor reversed the increase in MCF-7 cell invasion that was induced by human insulin in the *in vitro* Transwell migration and invasion assays. Therefore, insulin can promote breast cancer cell invasion and metastasis, which may be achieved by promoting endogenous miR-29a expression in breast cancer cells. These results suggest that miR-29a may be a new biomarker for the diagnosis of breast cancer, as well as a therapeutic target for the treatment of breast cancer.

Currently, two main insulin signal transduction pathways, namely, the Ras-MAPK pathway and PI3K-Akt/PKB pathway, are clearly involved in insulin-mediated promotion of breast cancer cell growth and proliferation [35], which were shown in supplementary material and Supplementary Figure 1. However, the mechanism by which miR-29a targets and participates in the Ras-MAPK and PI3K-Akt/PKB pathways needs to be studied further. In this study, after a miR-29a mimic was transfected into ER-positive breast cancer cells, p-ERK expression was significantly up-regulated, but Cdc42 and p85α expression was significantly down-regulated, and IGF-1R and ERK expression was not significantly altered in transfected cells compared with control cells. Moreover, after the miR-29a inhibitor was transfected into ER-positive breast cancer cells, p-ERK expression was down-regulated, but Cdc42 and p85α expression was restored, and small changes in IGF-1R and ERK expression were also observed. These results indicate that p-ERK, CDC42, and p85α are the target genes of miR-29a; however, miR-29a mainly acts through ERK phosphorylation to promote cell growth and proliferation. Shuming Gao et al. reported that IGF1 promoted angiogenesis. The IGF1 3′UTR functions as a ceRNA to promote angiogenesis by sponging the miR-29 family in osteosarcoma [22]. Zhao Z et al. reported that reduced miR-29a-3p expression is linked to cell proliferation and cell migration in gastric cancer [36]. The findings of Park SY and his partners provided new insights into the role of miRNAs in the p53 pathway. miR-29 family members (miR-29a, miR-29b and miR-29c) up-regulate p53 levels and induce apoptosis in a p53-dependent manner, and directly suppress p85 alpha (the regulatory subunit of PI3 kinase) and CDC42 (a Rho family GTPase), both of which negatively regulate p53 expression [23]. Based on these reports, we designed siRNA intervention experiments targeting IGF-1R, p85α and Cdc42. We found that miR-29a expression was down-regulated by IGF-1R-siRNA but not by p85α-siRNA or Cdc42-siRNA. We subsequently confirmed that IGF-1R is the upstream target gene of miR-29a, while CDC42 and p85α are the downstream target genes of miR-29a.

In this study, we demonstrated that insulin promotes miR-29a expression in breast cancer cells, regulates ER-positive breast cancer cell growth and invasion and participates in the insulin signaling pathway. We clarified that miR-29a-induced cell proliferation and metastasis acceleration occurred mainly through ERK phosphorylation and that IGF-1R is the upstream target gene of miR-29a, while CDC42 and p85α are the downstream target genes of miR-29a. We also elucidated the molecular mechanisms by which hyperinsulinemia promotes breast cancer occurrence and development and thus leads to a poor prognosis in breast cancer patients, and our results indicate that miR-29a plays an important role in breast cancer development and invasion.

## MATERIALS AND METHODS

### Cell culture

Human breast cancer cells, namely, MCF-7 and T47D cells, were obtained from the Cell Bank of the Chinese Academy of Science and maintained in Dulbecco's modified Eagle medium (DMEM, Gibco Carlsbad, CA, USA) supplemented with 10% fetal bovine serum (FBS, Gibco Carlsbad, CA, USA), 100 U/ml penicillin and 100 mg/ml streptomycin (Gibco Carlsbad, CA, USA) in a humidified incubator at 37°C in a 5% CO_2_ atmosphere.

### MTT assay

MCF-7 and T47D cells were incubated in culture medium supplemented with different concentrations of human insulin (0 IU/L, 5.0 IU/L, 20 IU/L, 50 IU/L, or 100 IU/L) for 48 h. Then, 10 μl of MTT solution (5 mg/ml, AMRESCO, USA) was added to each well, and the cells were incubated for an additional 4 h. Then, the supernatant was removed, and 100 μl of DMSO (AMRESCO, USA) was added to each well. When the colored materials had completely dissolved, the 96-well plate was read by an enzyme-linked immunoassay instrument (the absorbance wavelength was 490 nm; A value), and the cell proliferation rate was calculated as follows: cell proliferation rate = (A value of the experimental group - A value of the control group)/A value of the control group ×100. Determining the optimal insulin concentration was easily accomplished by comparing the cell proliferation rates of the groups treated with insulin at the above concentrations. MCF-7 cells were subsequently incubated in culture medium supplemented with the optimal concentration of human insulin for different lengths of time (0 h, 24 h, 48 h, 72 h, or 96 h). The optimal length of time for human insulin treatment was easily determined by comparing the cell proliferation rates of the groups treated for the indicated lengths of time.

### Quantitative real-time polymerase chain reaction (qRT-PCR)

Total RNA was extracted from cells frozen in TRIzol^®^ reagent, according to the manufacturer's protocol (Invitrogen, Paisley, UK), and stored at −80°C for preservation. RNA quality and quantity were determined by agarose gel electrophoresis and UV spectrophotometry.

To assess miR-29a expression, we performed qRT-PCR with a TaqMan Reverse-transcription Kit and TaqMan MicroRNA Assay Kit (Applied Biosystems, Foster City, CA, USA). U6 was used as a control. For mRNA analysis, we performed qRT-PCR with SYBR Green Master Mix (Roche Diagnostics GmbH, Mannheim, Germany); GAPDH was used as a control. The primer sequences for mature miR-29a, U6 snRNA and the miRNA target genes were synthesized by Shanghai Invitrogen Company and are shown in Supplementary Table 1. All experiments were performed in triplicate. The data were analyzed according to the comparative Ct (2^−ΔΔCt^) method.

### Oligonucleotide transfection

The miR-29a-5p mimic, inhibitor and negative-control molecules (scrambled control mimic and inhibitor) were synthesized and purified by the GenePharma Company (Shanghai, China). The sequences of these molecules are shown in Supplementary Table 1. They were transfected into cells at a final concentration of 50 nM using Lipofectamine-2000 transfection reagent (Invitrogen, Carlsbad, CA, USA), according to the manufacturer's protocol.

### Cell counting kit-8 (CCK-8) assay

Briefly, the cells were seeded in a 96-well plate at a density of 5 × 10^3^ cells per well in 100 μl of growth medium with high insulin and then incubated for 48 h at 37°C with 5% CO_2_. Then, the cells were transfected with 50 nM miR-29a-3p mimic, inhibitor or negative-control molecules using Lipofectamine-2000 (Invitrogen), according to the manufacturer's instructions. CCK-8 (Dojindo Laboratories, Kumamoto, Japan) was used to measure cellular growth, according to the manufacturer's instructions, and cell proliferation was assessed at different time points (0, 24, 48, and 72 h). The experiments were performed independently and in triplicate.

### Transwell migration and invasion assay

The migration and invasion potential of the transfected cells was evaluated by Transwell migration assay, which was performed using a Transwell chamber (BD Biosciences, Bedford, MA) with 8-μm pores. MCF-7 cells were grown to 70% confluence in normal medium and transfected with miR-29a-3p mimics or control mimics for 24 h, and additional MCF-7 cells were grown to 70% confluence in high-insulin medium and transfected with a miR-29a-3p inhibitor or control inhibitor for 24 h. In the migration assay, the cells were cultured in 200 ml of medium with 1% FBS in the upper chamber of a non-coated Transwell insert. Six hundred milliliters of medium with 10% FBS was placed in the lower chamber and used as a chemo-attractant to encourage cell migration. In the invasion assay, the upper chamber of the Transwell inserts was coated with 50 ml of 1.0 mg/ml Matrigel (Millipore, Billerica, MA, USA). Cells were subsequently plated in the upper chamber. After the cells had incubated for 24 h, the non-migrating or non-invading cells were gently removed with a cotton swab. All the invasive cells were stained using 0.1% crystal violet and were imaged and quantified by manual counting in five randomly selected areas.

### Western blotting

Total cell lysates were obtained from cells using 1× RIPA buffer (Beyotime, P0013B, Nanjing, China), according to the manufacturer's instructions. The samples were separated by SDS-PAGE and transferred to PVDF membranes. An immunoblot assay was employed for protein expression analysis. The following primary antibodies were purchased from Affinity Biosciences, OH, USA: an ERK1/2 antibody (AF0155), a phospho-ERK1/2 antibody (Thr202/Tyr204) (AF1015), a CDC42 antibody (DF6322), an AMPK alpha antibody (AF6423) a PI3-kinase p85-alpha antibody (AF6241), and a β-tubulin antibody (GS2006). The following antibodies were purchased from ProteinTech group, Inc., USA: a GAPDH antibody (60004-1), a CDK6 antibody (14052-1), and an IGF-1R-specific antibody (20254-1).

### Small interfering RNA (siRNA)-mediated IGF-1R knockdown

The cells were plated in 12-well plates at a density of 1.5 × 10^5^ cells/well 24 h before the first transfection. The siRNA sequences targeting human IGF-1R cDNA were designed and synthesized by Forevergen Biosciences (Guangzhou, China). A scrambled siRNA that could not target human IGF-1R cDNA was used as a negative control. The siRNA sequences are shown in Supplementary Table 2. The abovementioned siRNAs were transfected into MCF-7 cells using Lipofectamine 2000 (Invitrogen, CA, USA), according to the manufacturer's instructions.

### Statistical analyses

All experiments were performed at least three times. Variables were compared using a *t*-test or one-way ANOVA when appropriate. A *p* value < 0.05 was considered statistically significant. All analyses were performed using SPSS for Windows, version 18 (IBM, Armonk, NY, USA). The mean ± SD is displayed in each figure.

## SUPPLEMENTARY MATERIALS FIGURES AND TABLES



## Abbreviations

MTT: 3-(4,5-dimethylthiazol-2-yl)-2,5-diphenyl tetrazolium bromide
IGF-1: insulin-like growth factor 1
IRS-1: insulin receptor substrate 1
SHBG: sex hormone-binding globulin
IR: insulin receptor
TTP: tristetraprolin
DMSO: dimethyl sulfoxide
IGF-IR: insulin-like growth factor-1 receptor
CDK6: cyclin-dependent kinas 6
STATs: signal transducers and activators of transcription
HSP: heat-shock protein
PKC: protein kinase C
ER: estrogen receptor
PR: progesterone receptor
HER: human epidermal growth factor receptor
IHC: immunohistochemistry
BC: breast cancer
