# Supplementary material for: miR-29a regulated ER-positive breast cancer cell growth and invasion and is involved in the insulin signaling pathway
Source: Oncotarget. 2017 Mar 6;8(20):32566–75. doi: 10.18632/oncotarget.15928 (PMC5464809; doi:10.18632/oncotarget.15928)
Supplement: Supplementary file 1 [file oncotarget-08-32566-s001.pdf]

miR-29a regulated ER-positive breast cancer cell growth and invasion and is involved in the insulin signaling pathway

Supplementary Materials

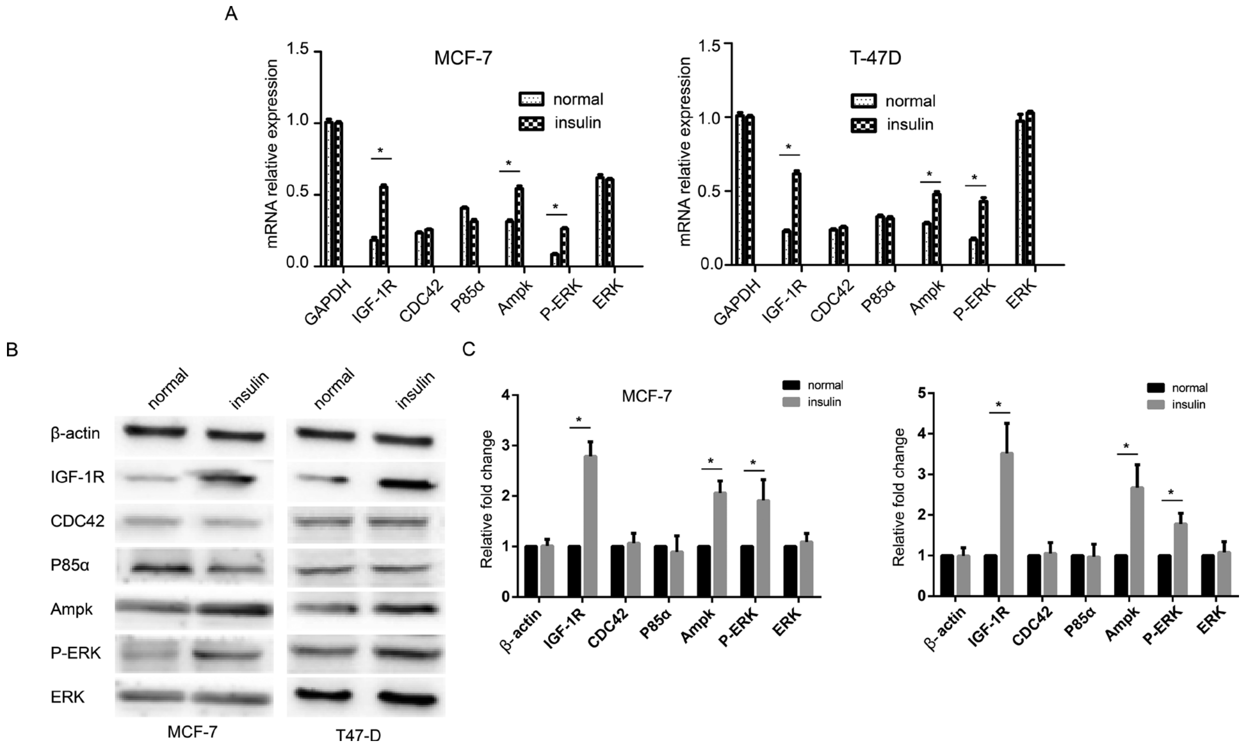

Supplementary Figure 1: Effect of insulin on the insulin signaling pathway in ER-positive breast cancer cells. IGF-1R, CDC42, P85α, Ampk, p-ERK and ERK expressions changes in mRNA and protein expression levels were detected by qRT-PCR (**A**) and western blotting (**B, C**) respectively. IGF-1R, AMPK and p-ERK expression upregulated in insulin groups. *$P < 0.05$

SUPPLEMENTARY MATERIALS AND METHODS

Effect of insulin on the insulin signaling pathway in ER-positive breast cancer cells

To investigate the effect of insulin on the insulin signaling pathway in breast cancer cells, we examined IGF-1R, CDC42, P85α, Ampk, p-ERK and ERK expression in MCF-7 and T47D cells cultured in normal medium or human insulin medium. Changes in mRNA and protein expression levels were detected by qRT-PCR (Supplementary Figure 1A) and western blotting (Supplementary Figure 1B) respectively. We found that IGF-1R, AMPK and p-ERK expression was significantly increased in the insulin group compared with the normal group and that P85α, CDC42, and ERK expression levels were not significantly different between the two groups. These results indicated that insulin mainly promotes ER-positive breast cancer cell growth and proliferation by upregulating IGF-1R, AMPK and p-ERK expression.

Supplementary Table 1: The primers for hsa-miR-29a and its target genes

U6	F primer	ATTGGAACGATACAGAGAAGAT
	R primer	GGAACGCTTCACGAATTT
hsa-miR-29a	F primer	TTTCCCTGGGTCTGGGCTGGGGC
	R primer	CCAGCCCAGACCCAGGGAAATGG
hsa-IGF-1R	F primer	ATGTCCAGGCCAAAACAGGAT
	R primer	CGGGTTCACAGAGGCATACA
hsa-p85-alpha	F primer	CCCGCAGAGGAAGGAAGC
	R primer	GTGCCACAGTCCTCTCACTG
hsa-CDC42	F primer	AGGCTATCCTAGCTGCCCTC
	R primer	GACGCAGAGGCTTTCAAACA
hsa-ERK	F primer	TGGAGCAGTATTACGACCCG
	R primer	TCCCTTGCTAGAGCTCACTG
hsa-P-ERK	F primer	CATCGCCGAAGCACCATTC
	R primer	CCCTTGCTAGAGCTCACTGTATT

Supplementary Table 2: Target sequences

miR-29a-5p mimic	Sense	ACUGAUUUCUUUUGGUGUUCAG
	Antisense	GAACACCAAAAGAAAUCAGUUU
miR-29a-5p inhibitor	CUGAACACCAAAAGAAAUCAGU	
microRNA inhibitor NC	CAG UACUUU UGU GUA GUA CAA	
miR-29a-5p negative control (microRNA mimic control)	Sense	UUC UCC GAA CGU GUC ACG UTT
	Antisense	ACG UGA CAC GUU CGG AGA ATT
IGR-1R siRNA	Sense	CCAAGCUAAACCGGCUAAA-dTdT
	Antisense	UUUAGCCGGUUUAGCUUGG-dTdT
CDC42 siRNA	Sense	CCAUCGGAAUAUGUACCGA-dTdT
	Antisense	UCGGUACAUAUUCCGAUGG-dTdT
P85α siRNA	Sense	GAAGCTCTCCAGACCATTT-dTdT
	Antisense	AAATGGTCTGGAGAGCTTCTT-dTdT
